# Comparative Analysis of Chemical Composition and Food Safety of Commercial Fish Sauces from Four Asian Countries

**DOI:** 10.3390/foods14173134

**Published:** 2025-09-08

**Authors:** Yu-Ru Huang, Pei-Chuan Wu, Chi-Jen Lo, Yi-Chen Lee, Yung-Hsiang Tsai

**Affiliations:** 1 Department of Food Science, National Ilan University, Ilan 260, Taiwan; 2 Metabolomics Core Laboratory, Healthy Aging Research Center, Chang Gung University, Taoyuan 333, Taiwan; chijenlo@mail.cgu.edu.tw; 3 Department of Seafood Science, National Kaohsiung University of Science and Technology, Kaohsiung 811213, Taiwan; lionlee@nkust.edu.tw (Y.-C.L.); yht@webmail.nkmu.edu.tw (Y.-H.T.)

**Keywords:** fish sauce, biogenic amines, preservatives, food labeling, food safety compliance, ^1^H-NMR metabolomics

## Abstract

This study evaluated chemical safety indicators in 38 commercial fish sauces from Thailand, South Korea, Taiwan, and Vietnam sold in Taiwan. We quantified key nitrogenous compounds, biogenic amines, preservatives, and sodium levels, and further characterized metabolite profiles using untargeted ^1^H nuclear magnetic resonance (^1^H-NMR) spectroscopy. Vietnamese fish sauces exhibited the highest total nitrogen content and lowest pH, indicating superior fermentation quality. Sodium concentrations ranged from 5037 to 12,637 mg/100 mL, and nearly 40% of products, particularly Thai and Korean, exceeded the permitted labeling tolerance (≤120%), highlighting substantial labeling inaccuracies. Preservative analysis revealed the unauthorized or excessive use of benzoates and sorbates in several samples, indicating regulatory non-compliance. Preservative analysis revealed that three of seven Taiwanese samples contained dehydroacetic acid above the regulatory limit of 1 g/kg, with sample C6 both mislabeled and showing the highest concentration (3.22 g/kg). Among the ten Vietnamese samples, two exceeded the permissible limits for combined preservative use, and samples D2–D5 contained triacetin, a non-listed food additive, in violation of current regulations. Notably, South Korean fish sauces contained histamine concentrations up to 539.85 ± 318.88 ppm, with several samples surpassing the Taiwanese regulatory limit of 400 ppm, raising significant food safety concerns. Metabolomic analysis differentiated products by country, with formic acid, acetate, branched-chain amino acids, and alanine contributing to the distinct profiles of Thai and Taiwanese fish sauces. Collectively, our results provide critical insights into the quality and safety of fish sauce products, highlighting the importance of monitoring biogenic amines and ensuring accurate labeling to comply with food safety regulations.

## 1. Introduction

Fish sauce (Yu-lu) is a brown liquid seasoning traditionally produced by fermenting fish, shrimp, or other marine ingredients with over 20% salt. It is rich in amino nitrogen (~20 g/L, with ~80% as amino acids) and imparts umami flavor to dishes [1]. Fish sauce consumption is popular in Southeast Asian countries, with over 80–90% of the population using it as a seasoning [2]. Fermentation time influences the taste and chemical composition of fish sauce. Umami flavor arises from peptides, amino acids, and nucleotides. During fermentation, complex metabolic pathways produce diverse volatile and non-volatile compounds that serve as key flavor substances and precursors. Market-based analyses, such as the Grand View Research: Fish Sauce Market Report, offer more specific insights into production, distribution, and consumption trends. The global fish sauce market size was valued at USD 2.07 billion in 2023 and is projected to grow at a CAGR of 5.1% from 2024 to 2030. The market is dominated by Southeast Asian producers, particularly Thailand and Vietnam, which are also the leading exporters. Major import markets include the United States, Japan, and South Korea, and global demand is growing due to the increasing popularity of Asian cuisine in Western countries [3].

With the increasing number of migrant workers and new immigrants, Southeast Asian cuisine has gradually become a regular dining option in Taiwan. Fish sauce is among the most commonly used seasonings in Southeast Asian cuisine. Fish sauce often contains histamine (Him), which is primarily associated with the free histidine content in the raw materials and the presence of Him-forming bacteria. These bacteria typically require extended fermentation periods to produce significant amounts of histamine. However, due to the prolonged fermentation involved in the production of fermented foods, the risk of histamine accumulation in such products can be considerable. Tsai et al. [4] reported Him levels of 45–1220 ppm in 12 fermented aquatic products from the Taiwanese market sourced from Southeast Asia and Taiwan. Zarei et al. [5] collected fish sauce (mahyaveh) from five different locations in Southern Iran, with an average Him content of 2662 mg/kg. Jiang et al. [6] collected 35 commercially available fish sauce products from three provinces in China. Concentrations of Him, putrescine (Put), cadaverine (Cad), and tyramine (Tyr), which were identified as major biogenic amines (BAs) in fish sauce, exceeded 100 mg/kg. Tryptamine (Trp), agmatine (Agm), and spermidine (Spm) were identified as minor BAs with concentrations < 25 mg/kg. Histamine poisoning typically presents within minutes to a few hours after consumption of contaminated food. Common symptoms include gastrointestinal discomfort (such as bloating and diarrhea), skin reactions (like itching and flushing), and respiratory issues (such as nasal congestion and shortness of breath). In more severe cases, cardiovascular and neurological symptoms—such as rapid heartbeat or headache—may occur. These effects usually resolve within 24 to 48 h, depending on individual sensitivity and the amount of histamine ingested [7].

Considering the increasing consumption and sale of fish sauce, this study aimed to evaluate the chemical composition, nitrogenous compounds, biogenic amines, and preservative contents of commercial fish sauce products available in the Taiwanese market. To further assess product quality and safety, metabolite profiles were examined using nuclear magnetic resonance (NMR) spectroscopy, followed by multivariate statistical analysis to reveal differences in metabolic composition across products from different countries.

## 2. Materials and Methods

### 2.1. Sample Collection

A total of 38 commercially available fish sauce products in glass bottles were collected from retail supermarkets, online shopping platforms, and grocery stores catering to foreign migrant workers in Taiwan, thereby covering all major distribution channels. All samples carried an expiration date as required by Taiwanese food labeling regulations, with validity periods ranging from January 2022 to December 2023. These products were made in Thailand (16 samples, labeled A1–A16), South Korea (5 samples, labeled B1–B5), Taiwan (7 samples, labeled C1–C7), and Vietnam (10 samples, labeled D1–D10). All samples were stored at 4 °C in the laboratory. Each fish sauce product was analyzed in triplicate. Appendix A Table A1 indicates the ingredients listed in the English labeling of fish sauce products.

### 2.2. Determination of Physical and Chemical Properties of Fish Sauce

The pH was determined using a pH meter (pH510; Eutech Instruments, Singapore). Water activity (Aw) was determined at 25 °C using an Aw analyzer (Rotronic HP23-AW-A; Rotronic AG, Bassersdorf, Switzerland). Fish sauce color was determined using the CR-400 Chroma Meter (Konica Minolta Sensing, Inc., Osaka, Japan) for lightness (L*), redness (a*), and yellowness (b*).

### 2.3. Determination of Salt Concentration

Total salt concentration was determined according to the general method of testing for heavy metal published by the Ministry of Health and Welfare of Taiwan (2014) (MOHWH0014.03) [8]. Briefly, 0.2 mL of the sample and 10 mL of concentrated 69% nitric acid were added to the digestion tube and digested for 80 min. The digestion solution was removed and diluted to 25 mL with deionized water to obtain a stock solution. Then, 50 μL of the sample stock solution was added and diluted to 25 mL with 1 N nitric acid, after which 1.25 mL of 10% cesium chloride was added. Absorbance was measured using a flame atomic absorption spectrophotometer (Polarized Zeeman Flame Atomic Absorption Spectrophotometer Z-6100; Hitachi, Tokyo, Japan) at 589 nm, and concentration was calculated from the standard curve.

### 2.4. Determination of Nitrogenous Compounds

#### 2.4.1. Total Volatile Basic Nitrogen (TVBN), Trimethylamine Oxide (TMAO), and Trimethylamine (TMA) Determination

Nitrogenous compounds in fish sauce were extracted using 7% trichloroacetic acid (TCA), following the method of Konosu et al. [9]. TVBN content was determined using the microdiffusion method described by Cobb et al. [10]. TMAO concentration was measured according to the method of Huang et al. [11], with slight modifications. Briefly, 1 mL of the TCA extract was mixed with toluene and KOH to extract TMA, followed by dehydration with anhydrous sodium sulfate and reaction with 0.02% picric acid for spectrophotometric analysis at 410 nm (Hitachi U-2000; Hitachi Co., Tokyo, Japan). Total TMA (TMA + TMAO) was obtained by reducing TMAO to TMA using TiCl_3_ in acidic conditions. The TMAO content was calculated by subtracting the initial TMA concentration from the total TMA.

#### 2.4.2. Determination of Total Nitrogen (TN), and Amino Nitrogen (AN)

TN content was determined using the Kjeldahl method [12] with digestion and distillation units (Model K-425/B-436 and K-350) (Buchi Labortechnik AG, Flawil, Switzerland). Formaldehyde nitrogen (FN), ammonia nitrogen (AM), and AN levels were determined according to the Thai Industrial Standards Institute [13]. AN was calculated based on the FN and AM contents as follows: AN content (g /L) = FN content (g /L) − AM content (g /L).

### 2.5. Determination of Biogenic Amine (BA) Content

TCA extracts of the fish sauce samples were derivatized with dansyl chloride, as previously described [11,14]. BAs were quantified using a high-performance liquid chromatography system (Young Lin, Anyang, Korea) equipped with a Model 9100 pump, Rheodyne Model 7125 manual injector, and Model 9160 photodiode array detector set at 254 nm. Chromatographic separation was achieved using the HiQsil C18 column (5 μm, 150 × 4.6 mm, i.d.; KYA Technology, Yokohama, Japan). The mobile phase began with an acetonitrile-to-water ratio of 50:50 (*v/v*) at a constant flow rate of 1.0 mL/min for the first 19 min. This was followed by a linear shift to 90:10 over 1 min, and finally returned to 50:50, held for an additional 10 min under the same flow conditions.

### 2.6. ^1^H NMR Spectroscopy and Spectral Analysis

The 20 μL of sample was extracted with 180 μL of extraction solution (deuterated methanol contained 0.03% trimethylsilyldiazomethane for the internal standard), evenly mixed for 4 min, and sonicated thrice for 5 min. The mixture was incubated at –40 °C for 1 h. The sample was centrifuged at 12,000× *g* for 30 min at 4 °C. Then, 180 μL of supernatant was transferred to a 3 mm NMR tube for NMR analysis. The NMR spectrometer contained the Bruker Avance III HD console combined with 14.1 T magnet (Bruker Biospin GmbH, Rheinstetten, Germany). It was equipped with a 5 mm inverse triple resonance probe (1H/13C/15N) with a z-axis gradient capable of automated tuning and matching the SampleJet system with a cooling rack to keep samples at 279 K. NMR data were acquired and processed automatically using the Topspin software and IconNMR program (version 3.2.2; Bruker Biospin GmbH, Rheinstetten, Germany). A ^1^H 1D nuclear Overhauser effect spectroscopy experiment with a solvent pre-saturation pulse sequence was used for data acquisition. A relaxation delay of 4 s and mixing time of 10 ms were applied for the nuclear Overhauser effect spectroscopy. A magnetic-field z-gradient was applied for 1 ms with an acquisition time of 2.7 s, and the spectral window was set at 20 ppm for the samples. A total of 128 transients were acquired, with 64 k data points for each sample. Line broadening was set to 0.3 Hz for processing, and zero filling by a factor of two was used to produce 128 k Fourier domain points.

NMR spectral data were processed and subjected to multivariate statistical analysis using SIMCA-P software (version 14.1; Umetrics, Umeå, Sweden), employing principal component analysis (PCA) and orthogonal partial least squares discriminant analysis (OPLS-DA). Identified metabolite signals were annotated through spectral matching with publicly available databases, including the Human Metabolome Database (HMDB; http://www.hmdb.ca/) and the Biological Magnetic Resonance Data Bank (BMRB; https://bmrb.io/).

### 2.7. Statistical Analyses

Statistical analyses were conducted using the SPSS software version 11.5 (SPSS Inc., Chicago, IL, USA). Repeated measures analysis of variance, Duncan’s multiple range, and Spearman’s correlation tests were used for statistical analyses. Statistical significance was set at *p* < 0.05.

## 3. Results and Discussion

### 3.1. Chemical Properties

In total, 38 fish sauce products, including 16 from Thailand (A1–A16), 5 from Korea (B1–B5), 7 from Taiwan (C1–C7), and 10 from Vietnam (D1–D10), available in the Taiwanese market were collected. As shown in Table 1, Korean fish sauce products exhibited a relatively high pH value of 5.80 ± 0.29 (5.60–6.13), which is significantly different from the products of the other three countries (*p* < 0.05). An elevated pH is typically associated with prolonged fermentation and increased protein degradation, which may lead to higher levels of biogenic amine formation, including histamine. Alkaline conditions can enhance the activity of certain histamine-producing bacteria, thereby increasing the risk of accumulation during extended fermentation processes. Accordingly, the higher histamine content observed in Korean fish sauce samples may be attributed to their longer fermentation duration and relatively higher pH values.

Comparatively, pH values of Thai and Vietnamese fish sauces were lower at 5.26 ± 0.28 (4.79–5.62) and 5.11 ± 0.42 (4.63–5.99), respectively. Notably, only B2 and C4 samples exhibited pH values > 6 (6.13 and 6.04, respectively). Cho et al. [15] reported that the pH of 15 Korean fish sauce samples was 5.56–6.47. Um and Park [16] also reported a pH of 5.51–6.14 (mean: 5.81) for seven Korean fish sauce samples.

Park et al. [17] analyzed fish sauce products from Southeast and East Asian countries and reported that the pH values of Thai, Vietnamese, and South Korean fish sauces were 5.63 ± 0.17, 5.75 ± 0.26, and 5.49 ± 0.45, respectively. Additionally, Nakano et al. [18] reported that the pH values of Thai and Vietnamese fish sauces were 4.89–5.22 and 4.91–5.10, respectively. Lopetcharat and Park [19] noted that most commercial fish sauces contain food additives, such as citric and sorbic acids, to adjust the pH during fermentation. In this study, Vietnamese fish sauces D2–D10, which contained citric acid, exhibited pH values of 4.63–5.57. Only sample D1, which did not contain citric acid, exhibited a higher pH value of 5.99, suggesting that the absence of citric acid contributed to its relatively higher pH value compared with those of other Vietnamese samples.

Table 1 shows that the fish sauces collected from Taiwan exhibited relatively high Aw values of 0.745–0.870, with an average of 0.813 ± 0.050. In contrast, the average Aw of products from the other three countries was 0.73–0.75. Notably, Aw values of the Taiwanese samples C4, C5, and C7 exceeded 0.85, indicating the necessity of careful storage. Aw = 0.85 is widely recognized as a microbiological safety threshold; below this level, the growth of most pathogenic bacteria is inhibited. The addition of salt during fish sauce fermentation reduces Aw. In this study, fish sauces collected from Taiwan and Vietnam exhibited a strong negative correlation between Aw and salt concentration (r = −0.88; *p* < 0.05).

Based on their sodium contents, fish sauces were ranked from highest to lowest as follows: Korea, Thailand, Vietnam, and Taiwan, with values of 11,546.30 ± 535.00, 11,018.18 ± 909.09, 9512.79 ± 1158.52, and 7968.11 ± 2319.58 mg/100 mL, respectively. These sodium levels corresponded to sodium chloride levels of 29.37 ± 1.36, 28.02 ± 2.31, 24.20 ± 2.95, and 20.27 ± 5.90 g/100 mL, respectively (Table 1). The order of the sodium content observed in this study is consistent with that reported by Yimdee and Wang [20].

Among the 38 collected fish sauce samples, 15 exhibited sodium content > 120% of the labeled nutritional value. Specifically, 6 out of 16 Thai samples (A5, A6, and A11–A14), all 5 Korean samples (B1–B5), 1 out of 7 Taiwanese samples (C7), and 3 out of 10 Vietnamese samples (D1, D8, and D10) did not comply with the allowable deviation of ≤120% from the labeled value, as stipulated by the regulations on nutrition labeling for packaged food products of the Ministry of Health and Welfare of Taiwan [21] (Table 2). The observed variation in sodium content among the fish sauce samples can be attributed to multiple factors. First, differences in manufacturing processes play a significant role, as producers may employ varying salt concentrations depending on preservation strategies, and recipes. Second, fermentation duration can influence sodium levels; longer fermentation periods often necessitate higher salt concentrations to inhibit the growth of spoilage microorganisms. Third, certain products may undergo dilution or contain added sweeteners, water, or flavorings, which can lower the final sodium concentration. Finally, regional taste preferences may also contribute to consumer expectations in different markets [22]. In countries such as Taiwan, compared with Southeast Asia, manufacturers can adjust salt levels to match local flavor profiles.

### 3.2. Nitrogenous Compounds of Fish Sauces

TVBN contents of the fish sauces from four countries, as shown in Table 1, varied considerably among the four countries, with the highest values observed in the Vietnamese sample (689.29 mg/100 g) and the lowest in the Thai sample (13.85 mg/100 g). Cho et al. [15] reported a TVBN content of 152.8–346.1 mg/100 g in 15 commercial South Korean anchovy fish sauce samples. Later, Um and Park [16] also reported that the TVBN content of seven South Korean fish sauce samples was 164.76–217.98 mg/100 g. Tsai et al. [4] reported that the TVBN content of 12 commercially available fish sauces in Taiwan was 51–270 mg/100 g, with a mean value of 194 ± 65 mg/100 g. TVBN content of both commercial and homemade fish sauces generally remained below 300 mg/100 g, except for 1 out of 15 South Korean anchovy fish sauce products that exhibited a TVBN content of 346.1 mg/100 g [15]. In this study, TVBN contents of 6/38 fish sauce samples, including Thai A1 (457.39 mg/100 g), A2 (331.10 mg/100 g), and A16 (310.03 mg/100 g) and Vietnamese D1 (500.33 mg/100 g), D8 (689.29 mg/100 g), and D9 (352.17 mg/100 g) samples, exceeded 300 mg/100 g.

Although TVBN is not an indicator of the fish sauce quality, its determination includes the analysis of amine compounds, such as TMA and dimethylamine (DMA). However, in this study, only TMA and trimethylamine N-oxide (TMAO) were quantified. The exclusion of DMA was due to this study’s focus on TMA/TMAO as representative freshness indicators. Nevertheless, DMA has been reported to contribute to spoilage-related odor and may react with nitrites under acidic conditions to form carcinogenic nitrosamines [23], which are classified as Group 2A carcinogens by the International Agency for Research on Cancer [24]. Future studies may benefit from including DMA quantification to provide a more comprehensive assessment of volatile amine profiles and potential safety concerns in fish sauce products.

As shown in Table 1, TMA contents in the fish sauce samples of Thailand, South Korea, Taiwan, and Vietnam were 7.80–49.28, 12.73–72.14, 19.03–32.17, and 2.05–66.66 mg/100 g, respectively. TMAO content in all samples was ND–69.80 mg/100 g. Final TMA and TMAO contents in the products were influenced by various factors, such as fish species, body size, fishing season, and fermentation endpoint. It is worth noting that TVBN, TMAO, and TMA are widely recognized as indicators of raw material freshness in fish and fish-derived products. TVBN reflects the accumulation of volatile nitrogenous compounds generated during microbial and enzymatic protein degradation, with elevated values generally associated with poorer raw material quality [25]. TMAO, which is naturally abundant in marine fish, is enzymatically or microbially reduced to TMA during spoilage; therefore, higher TMA levels are indicative of reduced freshness and quality. In fish sauce, however, the levels of these compounds depend not only on the initial freshness of raw materials but also on fermentation processes and duration, which further contribute to their variability [26].

### 3.3. Total Nitrogen (TN) and Amino Nitrogen (AN)

TN includes amino and non-amino nitrogenous compounds, such as free amino acids, nucleotides, peptides, ammonia, urea, and TMAO. Almost 60–80% of the nitrogenous compounds are amino acids in fish sauce. TN content is related to the fish sauce quality [27]. Table 3 shows that the average TN content in all fish sauces, from highest to lowest, was as follows: 2.45–56.40 (22.00 ± 16.46), 5.93–20.09 (13.32 ± 4.70), 9.01–13.02 (10.70 ± 2.03), and 1.80–8.19 (4.77 ± 2.54) g/L for Vietnam, Thailand, South Korea, and Taiwan, respectively. According to the Thai Industrial Standards Institute (TISI) [13] classification, fish sauce products with TN contents of 15–20 g/L are classified as Grade 2, whereas those with TN contents > 20 g/L are classified as Grade 1. Among the 16 Thai fish sauce samples analyzed in this study, only sample A1 exhibited a TN content of 20.09 g/L, serving as a Grade 1 product. An additional six samples (A2–A5, A12, and A16) were classified as Grade 2, with TN contents of 15.19–19.81 g/L, whereas the remaining nine samples exhibited TN contents < 15 g/L.

The Korea Agency for Technology and Standards [28] stipulates that standard fish sauce must exhibit TN content ≥ 12 g/L and AN content ≥ 6 g/L. Premium fish sauce requires TN content ≥ 16 g/L and AN content ≥ 9 g/L. Seasoned fish sauce is defined by TN content ≥ 5 g/L. Here, TN and AN contents of the B1 and B4 Korean fish sauces were 12.81–13.02 and 6.02–8.32 g/L, respectively. Therefore, B1 and B4 are classified as standard fish sauces.

Fish sauce products in Vietnam labeled as “Nước Mắm” are 100% pure fish sauces, whereas seasoned fish sauce is referred to as “Nước Chấm.” Taiwan and Vietnam have no regulations on fish sauce quality. Among the 10 Vietnamese fish sauce samples analyzed in this study, D1–D4, D6, D8, and D10 were labeled as 100% pure fish sauce. D7 was labeled as seasoned fish sauce. D5 and D9 did not indicate the grade of the fish sauce. D3 and D9 were labeled as containing more than 10 g/L and 35 g/L of total nitrogen (TN), respectively. The analytical results were consistent with the product. In contrast, lower TN in other countries, such as Taiwan, may be related to shorter fermentation and dilution with water, sugar, or flavorings to match local taste preferences.

Fish sauce samples of Taiwan and Vietnam were classified according to the fish sauce grading definitions of the Korean Agency for Technology and Standards. Fish sauces from Taiwan (C1, C2, C6, and C7) were classified as seasoned fish sauces, whereas the TN contents of C3–C5 were below 5 g/L; therefore, they were not classified as standard fish sauces. Fish sauce samples of Vietnam (D3–D5) were classified as standard fish sauces. D1, D6, and D8–D10 were classified as premium fish sauces, whereas D2 was classified as a seasoned fish sauce. TN content of D7 was below 5 g/L; therefore, it was not classified as a standard fish sauce.

The AN content of fish sauces, from highest to lowest, was as follows: 13.97 ± 10.70 (0.74–32.56), 7.62 ± 2.67 (3.50–12.46), 5.97 ± 2.48 (2.15–8.32), and 3.28 ± 2.57 (0.97–8.41) g/L for Vietnam, Thailand, South Korea, and Taiwan, respectively (Table 3). The content of AN in Korean fish sauce was 2.15–8.32 g/L, which was similar to the AN contents of commercially available Korean anchovy fish sauce (4.79–9.25 g/L) and sand lance fish sauce (4.34–10.07 g/L) reported by Cho et al. [29]. However, the AN content was slightly lower than that (8.19–14.21 g/L) reported by Joung and Min [30]. In this study, based on the TN and AN contents, fish sauce samples of different countries were ranked in the following descending order: Vietnam, Thailand, Korea, and Taiwan (Table 3).

### 3.4. Biogenic Amine (Bas)

Him content in the fish sauce products, from highest to lowest, was as follows: 539.85 ± 318.88 (192.11–1046.05 ppm), 194.08 ± 137.49 (ND–511.39 ppm), 150.16 ± 136.97 (26.09–514.54 ppm), and 60.40 ± 55.68 (6.53–116.96 ppm) for Korea, Vietnam, Thailand, and Taiwan, respectively (Table 4). All seven fish sauce products from Taiwan complied with the regulatory standards, whereas samples A12, B1–B4, and D1 from other countries exceeded the Taiwanese regulatory limit of 400 ppm. Him concentration in Korean fish sauce (539 ± 318 ppm) in this study was similar to that of the Korean fish sauce (443 ppm) [30]. A survey of 12 commercially available Korean fish sauce products revealed that only 1 product complied with the Codex standard, indicating that Him levels in fish sauce should be <400 ppm. However, Korea currently has no regulations on Him levels [30]. Moon et al. [31] also reported that all 15 anchovy fish sauces exhibited Him levels > 500 ppm, with 4 products exhibiting Him levels > 1000 ppm. Additionally, six sand lance fish sauce products contained Him levels > 500 ppm. Therefore, the BA content of Korean fish sauce products should be carefully monitored.

Put (putrescine) and Cad (cadaverine) exhibit relatively low intrinsic toxicity but inhibit the activity of enzymes involved in the metabolism of Him and Tyr, thereby increasing the levels of these BAs and exacerbating discomfort in the human body. Put content of the fish sauces, from highest to lowest, was as follows: 44.63 ± 44.12 (range: 9.75–161.67), 33.55 ± 13.98 (range: 19.53–53.42), 24.35 ± 26.94 (range: ND–91.56), and 15.91 ± 19.73 (range: ND–52.87) ppm for Thailand, South Korea, Vietnam, and Taiwan, respectively. Only sample A12 exhibited Put content > 100 ppm (160 ppm), whereas no Put was detected in samples C4 and D7 (Table 4). Cad content of fish sauce samples, from highest to lowest, was as follows: 94.95 ± 84.26 (range: 28.24–360.06), 53.77 ± 35.20 (range: 1.68–116.22), 44.34 ± 15.73 (range: 20.60–64.85), and 23.53 ± 40.38 (range: ND–97.00) ppm for Thailand, Vietnam, South Korea, and Taiwan, respectively. Cad content in Thai fish sauce was significantly higher than those in the other three countries, with sample A12 showing the highest Cad content of 360.06 ppm. High levels of Him, Put, and Cad observed in sample A12 indicate a risk of allergic reactions. Kim et al. [32] reported that most fish sauce products sold in Korea meet the basic physicochemical standards, such as TN content, but contain high Him levels (419.10–1025.50 mg/kg). Kimura et al. [33] isolated high histamine-producing strains from fermented products, some of which were salt-tolerant or halophilic bacteria capable of producing biogenic amines (BAs) under low pH (5.8) and oxygen-restricted conditions. For example, the halophilic lactic acid bacterium *Tetragenococcus muriaticus* can form histamine even in the presence of 20% NaCl. These findings underscore that histamine formation in fish sauce is not entirely prevented by high salt concentrations, and that careful control of raw material quality, fermentation conditions, and storage is essential to minimize food safety risks.

Kang [34] noted that the addition of fish sauce during kimchi fermentation increases Him levels in the final product. Tran et al. [35] reported that Him poisoning from fish sauce is rare due to the relatively small amount of food consumed per meal. However, fish sauce with high Him content cannot be imported for sale in Europe or the United States. According to the U.S. Food and Drug Administration (FDA), the level of histamine considered hazardous to health is 50 ppm [36]. In the European Union, Regulation (EU) No 1019/2013 sets a maximum limit of 400 mg/kg for histamine in fish sauce produced by fermentation [37]. These regulations reflect the strict maximum residue limits (MRLs) enforced in both major markets to ensure consumer safety.

### 3.5. Preservatives

As shown in Table 5, among the 38 fish sauce samples collected for this study, only products from Taiwan (C1, C2, C4, and C5) and Vietnam (D2–5, D7, and D10) indicated the use of preservatives (benzoic acid, sodium benzoate, and potassium sorbate) on their product labels. Taiwan adopts a positive list approach to regulate preservatives in food products. Any food imported, manufactured for sale, or sold must contain only specified permitted preservatives and their amounts must not exceed the maximum permitted level. In this study, we tested five acid-type preservatives: benzoic, sorbic, dehydroacetic, salicylic, and p-hydroxybenzoic acids. Dehydroacetic and salicylic acids are not allowed in sauce products and were not detected in any of the 38 fish sauce samples. Similarly, p-hydroxybenzoic acid was not detected in the samples.

In accordance with Taiwanese regulations, the permitted limits for sorbic and benzoic acids in sauce products are below 1 g/kg [21]. As shown in Table 5, three of the seven Taiwanese fish sauce products were non-compliant. Sorbic acid levels in C1, C2, and C6 were 3.07, 1.69, and 3.22 g/kg, respectively. Notably, C6 was labeled as “no preservatives added” but contained sorbic acid at the highest concentration among all samples. Among the ten Vietnamese fish sauce products, two were non-compliant (D7 and D10). D7 and D10 contained sorbic and benzoic acids, respectively. D10 exceeded the regulatory limit of sorbic acid (1 g/kg) at a concentration of 1.12 g/kg. Although the preservative levels in D7 did not exceed 1 g/kg for either preservative, when preservatives were used in combination, the regulation requires that the sum of the ratios of each preservative added amount to its permitted limit (added amount/maximum permitted limit) should not exceed 1. The sum for D7 was 1.34, making it a non-compliant product.

### 3.6. Metabolomic Analysis of Fish Sauce via ^1^H-NMR Spectroscopy

This study used ^1^H-NMR spectroscopy to analyze the metabolites in commercial fish sauce products from Thailand, South Korea, Taiwan, and Vietnam available in the Taiwanese market. ^1^H-NMR metabolomics enables the high-throughput non-destructive identification of various compounds and comprehensive identification of different known and unknown compounds with high specificity and reliability. PCA and OPLS-DA were used for classification, with variable importance in projection scores used to identify the key differentiating metabolites. In this study, the PCA score plot revealed limited separation between groups. Therefore, we proceeded with OPLS-DA, which effectively identified differentiating metabolites that were not clearly distinguishable in the PCA model. Only metabolites contributing to the sample grouping were quantified. This study focused only on metabolites exhibiting major differences in their NMR spectral signals. Signals that did not contribute to the sample grouping (as determined by OPLS-DA) were not quantified. The primary contributors to group differentiation include three amino acids (alanine [Ala], valine [Val], and glycine [Gly]), three organic bases (betaine, TMA, and DMA), two acids (acetate and lactate), dimethyl sulfide (DMS), and glucose. Notably, free amino acids were the predominant compounds detected within the chemical shift range 2–4 ppm. DMS is formed by the enzymatic degradation of cysteine and methionine [38]. It is a common sulfur-containing compound in fish sauce with an odor threshold value of 0.3 ppb. Owing to its low threshold, DMS contributes to fecal odor, making it a primary factor affecting the fish sauce aroma [2,39,40]. DMS content of the fish sauce samples from the four countries, in descending order, was as follows: 2.99–6.37 (4.15 ± 1.44), 0.33–10.52 (3.80 ± 3.63), 1.74–8.49 (3.44 ± 1.57), and 0.43–2.33 (0.99 ± 0.61) mg/100 mL for Korea, Vietnam, Thailand, and Taiwan, respectively (Table 6). All samples exhibited a strong fecal odor due to DMS (odor activity value > 10,000), with samples A1, B1, D1, and D2 exhibiting particularly high DMS concentrations, with odor activity values > 200,000. Conversely, the lower DMS levels in Taiwanese products may be linked to shorter fermentation durations, dilution with non-fermented ingredients, and the use of raw fish species with relatively low sulfur amino acid content. Previous studies have demonstrated that targeted microbial interventions can influence sulfur compound profiles; notably, Udomsil et al. [41] reported that strains of *Tetragenococcus halophilus* not only enhanced glutamate levels but also reduced volatile sulfur compounds responsible for fecal odors, thereby improving the overall flavor quality of fish sauce.

Among the samples analyzed, only A1, C2, C3, D1, and D3 exhibited glucose concentrations exceeding the threshold, with taste activity values (TAVs) of 1.08, 1.33, 1.20, 1.84, and 3.42, respectively (Table 7). The sweetness provided by glucose was most pronounced in D3. As some fish sauce manufacturers add sugars or sweeteners after fermentation, we could not determine the correlation between glucose and its metabolic products in the fish sauce products collected in this study. However, statistical analysis revealed that, among the 38 samples, metabolites acetate, lactate, TMA, and DMA exhibited significant positive correlations (r > 0.7; *p* < 0.001). Taiwanese fish sauce was significantly different from those of the other three countries that exhibited the lowest concentrations of all metabolites (*p <* 0.05). Among these metabolites, acetate, TMA, and DMA are odor-active compounds with odor threshold values of 22,000, 0.47, and 84.6 ppb, respectively [42,43]. All samples exhibited noticeable sourness due to acetate and a fishy odor due to TMA and DMA (Table 7).

In this study, we quantified the three most abundant amino acids in fish sauce. Ala and Gly are sweet-tasting amino acids with taste thresholds of 60 and 130 mg/100 mL, respectively, whereas Val is a bitter-tasting amino acid with a threshold of 40 mg/100 mL [45]. TAV was calculated by dividing the amino acid content of each sample by its respective threshold to assess its contribution to the taste profile of fish sauce. As shown in Table 6, Ala content, in descending order, was as follows: 44.04 ± 31.76 (0.81–96.96), 29.04 ± 12.19 (15.93–48.70), 28.68 ± 14.90 (4.43–73.63), and 10.61 ± 6.52 (1.84–20.53) mg/100 mL for Vietnam, Korea, Thailand, and Taiwan, respectively. Ala content of Taiwanese fish sauce was significantly lower than those of the other three country sauces (*p* < 0.05). Gly content was not significantly different among the four countries (*p* > 0.05). Samples with Gly TAV > 1 included A1, A2, D2, and D6, with A1 exhibiting the highest TAV of 2.19. However, Gly contents of the Korean and Taiwanese fish sauce samples were below the taste threshold, indicating that the sweetness imparted by Gly was not perceptible. Only samples A1, B1, D1, and D2 exhibited sufficiently high Val concentrations to impart bitterness. However, the presence of sweet-tasting amino acids and sweeteners possibly masked the bitter taste, making it less perceptible (Table 7).

**Figure 1:**
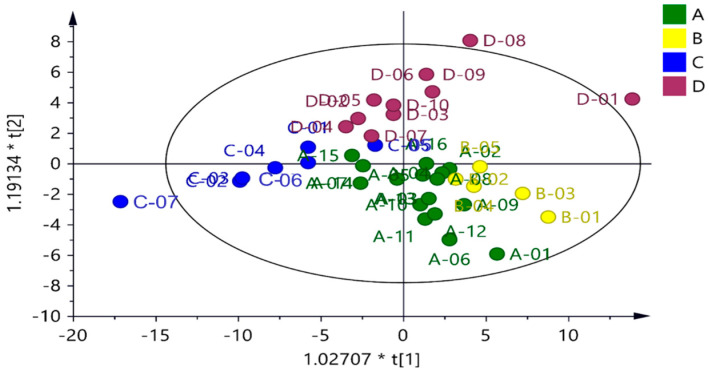
Orthogonal partial least squares discriminant analysis (OPLS-DA) of the fish sauce samples of different countries. Score plot derived from the ^1^H-NMR spectra of the fish sauce samples of different countries (A: Thai, B: Korea, C: Taiwan, and D: Vietnam).

**Figure 2:**
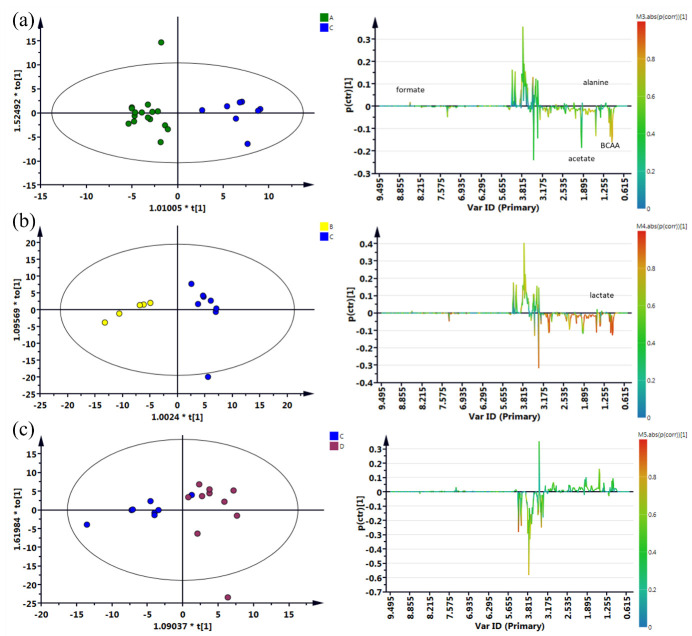
OPLS-DA score plots derived from the ^1^H-NMR spectra of fish sauce samples to discriminate their geographical origin. (**a**) C: Taiwanese/A: Tai fish sauce. (**b**) C: Taiwanese/B: Korean fish sauce. (**c**) C: Taiwanese/D: Vietnamese fish sauce.

As shown in Figure 1, metabolite grouping of fish sauce samples by country revealed that the Thai and Korean fish sauces exhibited similar metabolic profiles, leading to poor separation. McKelvie et al. [46] reported that overlapping resonances of amino acids and sugars in the samples made it difficult to accurately distinguish the individual metabolite peaks in the 3.0–4.5 ppm region of the ^1^H NMR spectrum. Here, C7, D1, and D8 exhibited more distinct metabolic differences, whereas C5 and D7 exhibited similar metabolite compositions, with a slight overlap. As shown in Figure 2a,b, Thai and Korean fish sauces showed good separation from the Taiwanese samples. However, overlap between Taiwanese samples C5 and D7 led to a weaker separation (Figure 2c). Key metabolites that differentiate Taiwan from other countries were identified. Thai fish sauce was distinguished by formic acid (δ 8.44 ppm), acetate (δ 1.99 ppm), branched-chain amino acids (δ 0.94–0.99 ppm), and Ala (δ 1.39 ppm), which may be linked to the use of protein-rich fish species and extended fermentation times. Korean fish sauce was mainly distinguished by elevated lactic acid (δ 1.33 ppm), possibly reflecting a greater contribution of lactic acid bacteria activity during fermentation. Vietnamese fish sauces, with their high total nitrogen content and low pH, suggest more intensive proteolysis and accumulation of amino acid-derived compounds, likely due to longer fermentation periods and minimal dilution before bottling. Taiwanese fish sauces, in contrast, showed the lowest concentrations of most key metabolites, which may be related to shorter fermentation durations or different raw material compositions.

These differences in metabolite composition collectively reflect variations in raw fermentation materials and production parameters across countries. The analytical approach used in this study also highlights the broader applicability of ^1^H-NMR spectroscopy in seafood quality assessment. For example, Ciampa et al. (2022) [47] demonstrated that ^1^H-NMR can be applied to determine both trimethylamine (TMA) and K-index values in seafood through a simple acid extraction procedure, eliminating the need for filtration, derivatization, or other complex sample preparations. Such methodology could be extended to a wide range of seafood products, both raw and cooked, offering a rapid non-destructive tool for compositional and freshness evaluation [48].

## 4. Conclusions

In conclusion, this study comprehensively analyzed the chemical properties and metabolite compositions of the fish sauce samples of Thailand, South Korea, Taiwan, and Vietnam available in the Taiwanese market. Significant differences in pH, Aw, sodium, nitrogenous compound, biogenic amine (BA), and preservative contents were observed among the countries. The South Korean fish sauce exhibited the highest pH and histamine levels, raising potential food safety concerns, whereas the samples from Taiwan exhibited the highest Aw. Vietnamese fish sauce exhibited the highest TN and AN contents, indicating its extensive fermentation. Importantly, the relatively high histamine and other BA contents in some products highlight the necessity of strict monitoring, as excessive intake may pose health risks. Salt/sodium levels also varied considerably across countries, suggesting that clearer labeling and control of sodium content should be considered to reduce dietary risks. Additionally, preservative analysis revealed that some fish sauces did not comply with regulatory standards, emphasizing the need for stringent monitoring and enforcement.

Based on these findings, we recommend conducting more frequent and targeted inspections of both imported and domestically produced fish sauce products. These inspections should focus on biogenic amine levels, especially histamine, enforce compliance with preservative regulations, and establish harmonized labeling tolerance limits consistent with international standards. Moreover, integrating sodium content into labeling and regulatory oversight could help mitigate health risks and improve consumer awareness. Metabolomic analysis via ^1^H-NMR revealed significant differences in the amino acid, organic acid, and sulfur-containing compound contents of fish sauces from different countries, which affected their flavor and quality. The main limitation is that only one retail unit per brand was analyzed, which may not capture batch-to-batch variability. Overall, this study provides critical insights into the quality and safety of fish sauce products, highlighting the importance of monitoring biogenic amines, controlling sodium levels, and ensuring accurate labeling to comply with food safety regulations. These results offer valuable evidence to strengthen food regulation, enhance quality control, and promote safer production and consumption of fish sauces.

## Figures and Tables

**Table 1:** General physical and chemical analyses of the fish sauces of various countries.

	No. of Sample	pH	Aw	Na (mg/100 mL)	TVBN ^1^ (mg/100 g)	TMA ^2^ (mg/100 g)	TMAO ^3^ (mg/100 g)
Thailand	16	4.79–5.62 ^4^ (5.26 ± 0.28) ^b,5^	0.701–0.841 (0.731 ± 0.033) ^b^	8621.96–12,637.12 (11,018.18 ± 909.09) ^a^	13.85–457.03 (199.16 ± 106.93) ^ab^	7.80–49.28 (23.78 ± 10.96) ^b^	ND ^6^–61.64 (33.37 ± 16.64) ^a^
Korea	5	5.60–6.13 (5.80 ± 0.29) ^a^	0.735–0.767 (0.747 ± 0.012) ^b^	10,323.69–12,308.68 (11,546.30 ± 535.00) ^a^	147.49–236.29 (186.66 ± 38.87) ^ab^	12.73–72.14 (42.93 ± 21.94) ^a^	ND–41.89 (18.15 ± 15.30)
Taiwan	7	4.51–6.04 (5.50 ± 0.59 ) ^ab^	0.745–0.870 (0.813 ± 0.050) ^a^	5037.55–11,417.20 (7968.11 ± 2319.58) ^b^	38.95–117.39 (66.45 ± 29.80) ^b^	19.03–32.17 (27.44 ± 4.78) ^ab^	1.64–47.64 ^ab^ (20.85 ± 14.49)
Vietnam	10	4.63–5.99 (5.11 ± 0.42) ^b^	0.674–0.822 (0.754 ± 0.056) ^b^	8621.96–11,334.49 (9512.79 ± 1158.52) ^c^	26.65–689.29 (253.71 ± 205.53) ^a^	2.05–66.66 (31.57 ± 20.29) ^ab^	ND–69.80 (13.55 ± 21.79) ^b^

^1^ TVBN—total volatile basic nitrogen; ^2^ TMA—trimethylamine; ^3^ TMAO—trimethylamine N-oxide; ^4^ Values are presented as concentration ranges (minimum–maximum); ^5^ mean ± SD values in the same column with different letters are statistically different (*p* < 0.05); ^6^ ND—not detected (TMAO content < 0.75 ppm).

**Table 2:** Sodium content of fish sauce samples exceeding the allowable labeling deviation.

Code ^1^	Label Values (mg/100 mL)	Laboratory Values (mg/100 mL)	Tolerance Limit (%) ^2^
A5	7400	10,791.60	146
A6	7400	11,542.32	156
A11	7990	11,041.84	138
A12	6900	12,167.92	176
A13	8850	11,135.68	126
A14	7300	10,134.72	139
B1	6821	12,308.68	180
B2	6344	11,334.49	179
B3	8955	11,158.07	125
B4	6821	11,886.40	174
B5	7579	11,043.86	146
C7	3950	5037.55	128
D1	8100	11,229.52	139
D8	6064	10,365.73	171
D10	6800	8621.96	127

^1^ A, Thailand; B, Korea; C, Taiwan; D, Vietnam; ^2^ comparing label and laboratory values: tolerance limit (%) = (laboratory values/label values) × 100%.

**Table 3:** Contents (g/L) of total nitrogen (TN), and amino nitrogen (AN) in the fish sauces of various countries.

Country	TN ^1^	AN ^2^
Thailand	5.93–20.09 ^3^ (13.32 ± 4.70) ^ab,4^	3.50–12.46 (7.62 ± 2.67) ^b^
Korea	9.01–13.02 (10.70 ± 2.03) ^b^	2.15–8.32 (5.97 ± 2.48) ^b^
Taiwan	1.80–8.19 (4.77 ± 2.54) ^b^	0.97–8.41 (3.28 ± 2.57) ^b^
Vietnam	2.45–56.40 (22.00 ± 16.46) ^a^	0.74–32.56 (13.97 ± 10.70) ^a^

^1^ TN—total nitrogen; ^2^ AN—amino nitrogen. ^3^ Values are presented as concentration ranges (minimum–maximum). ^4^ Mean ± SD values in the same column with different letters are statistically different (*p* < 0.05).

**Table 4:** Contents of various biogenic amines in the fish sauces of various countries.

Country	No. of Sample	Trp ^1^	Pea ^2^	Put ^3^	Cad ^4^	Him ^5^	Tyr ^6^	Spd ^7^	Spe ^8^
		Contents of Biogenic Amine (ppm)							
Thailand	16	5.04–63.65 ^9^ (18.96 ± 14.89) ^b,10^	1.52–25.57 (8.03 ± 6.34) ^b^	9.75–161.67 (44.63 ± 44.12) ^a^	28.24–360.06 (94.95 ± 84.26) ^a^	26.09–514.54 (150.16 ± 136.97) ^b^	6.21–86.32 (32.57 ± 20.04) ^a^	ND–11.68 (1.01 ± 3.00) ^a^	ND–0.36 (0.05 ± 0.12) ^b^
Korea	5	88.59–219.29 (127.46 ± 55.72) ^a^	17.37–37.72 (26.54 ± 8.28) ^a^	19.50–53.42 (33.55 ± 13.98) ^a^	20.60–64.85 (44.34 ± 15.73) ^ab^	192.11–1046.05 (539.85 ± 318.88) ^a^	14.02–55.00 (35.25 ± 15.06) ^a^	ND (ND) ^a,11^	ND (ND) ^b^
Taiwan	7	1.88–11.82 (5.21 ± 3.70) ^c^	0.11–32.37 (6.63 ± 11.56) ^b^	ND–52.87 (15.91 ± 19.73) ^a^	ND–97.00 (23.53 ± 40.38) ^b^	2.72–132.71 (60.40 ± 55.68) ^b^	6.53–116.96 (41.26 ± 41.97) ^a^	ND (ND) ^a^	ND–7.16 (2.76 ± 3.09) ^a^
Vietnam	10	0.53–137.95 (42.58 ± 37.14) ^b^	ND–99.56 (18.03 ± 29.04) ^ab^	ND–91.56 (24.35 ± 26.94) ^a^	1.68–116.22 (53.77 ± 35.20) ^ab^	ND–511.39 (194.08 ± 137.49) ^b^	2.74–34.86 (15.68 ± 9.57) ^a^	ND–88.16 (12.89 ± 27.99) ^a^	ND (ND) ^b^

^1^ Trp—tryptamine; ^2^ Pea—phenylethylamine; ^3^ Put—putrescine; ^4^ Cad—cadaverine; ^5^ Him—histamine; ^6^ Tyr—tyramine; ^7^ Spd—spermidine; ^8^ Spe—spermine; ^9^ values are presented as concentration ranges (minimum–maximum); ^10^ lowercase letters indicate the comparisons among different countries (*p* < 0.05); ^11^ ND—not detected (amine content < 0.05 ppm).

**Table 5:** Contents of various preservatives in fish sauces.

Sample	Product Label	Benzoic Acid (g/kg)	Sorbic Acid (g/kg)
C1	Sorbic acid	ND ^1^	3.07 ^a^
C2	Sorbic acid	ND	1.69 ^a^
C4	Sorbic acid	ND	ND ^b^
C5	Benzoic acid	0.53	ND
C6	None	ND	3.22 ^ac^
D2	Benzoic and sorbic acids	0.54	ND ^b^
D3	Benzoic and sorbic acids	0.58	0.11
D4	Benzoic and sorbic acids	0.50	ND
D5	Benzoic acid	0.59	ND
D7	Benzoic and sorbic acids	0.46	0.87
D10	Benzoic and sorbic acids	0.39	1.12 ^a^

C, Taiwan; D, Vietnam. ^1^ ND—not detected (preservative content < 0.02 g/kg). ^a^ Laboratory values exceed the Taiwan regulation limits within 1 g/kg. ^b^ Preservatives do not conform to the product label. ^c^ Sample does not have a product label but contains preservatives.

**Table 6:** Comparison of the compositions of the major metabolites in fish sauces from various countries using nuclear magnetic resonance (NMR) spectroscopy (mg/100 mL).

	Thailand	Korea	Taiwan	Vietnam
Glucose	425.50 ± 241.06 ^ab,4^ (49.82–932.22) ^5^	34.80 ± 27.49 ^b^ (3.35–64.10)	762.98 ± 479.83 ^a^ (3.14–1580.92)	389.40 ± 906.02 ^ab^ (2.12–2940.64)
Betaine	4.16 ± 2.14 ^ab^ (0.79–10.62)	5.41 ± 2.87 ^a^ (1.78–9.78)	1.82 ± 1.13 ^b^ (0.49–4.19)	4.50 ± 4.33 ^ab^ (0.13–12.16)
TMA ^1^	1.14 ± 0.54 ^bc^ (0.35–2.66)	2.57 ± 0.83 ^a^ (1.49–3.70)	0.22 ± 0.16 ^c^ (0.03–0.48)	1.49 ± 1.90 ^b^ (0.04–6.18)
DMA ^2^	0.56 ± 0.39 ^a^ (0.11–1.68)	0.30 ± 0.14 ^ab^ (0.10–0.45)	0.12 ± 0.07 ^b^ (0.03–0.25)	0.32 ± 0.46 ^ab^ (0.02–1.50)
Acetate	37.89 ± 18.81 ^a^ (6.03–94.22)	39.92 ± 17.50 ^a^ (17.81–60.01)	6.87 ± 2.92 ^b^ (3.05–11.83)	29.96 ± 33.38 ^a^ (4.22–98.34)
Alanine	28.68 ± 14.90 ^ab^ (4.43–73.63)	29.04 ± 12.19 ^ab^ (15.93–48.70)	10.61 ± 6.52 ^b^ (1.84–20.53)	44.04 ± 31.76 ^a^ (0.81–96.96)
Valine	38.03 ± 18.74 ^a^ (6.13–93.53)	37.78 ± 13.08 ^a^ (28.44–60.08)	9.08 ± 5.58 ^b^ (2.90–19.36)	37.72 ± 39.17 ^a^ (0.79–115.13)
Lactate	20.26 ± 9.95 ^ab^ (4.20–48.22)	15.23 ± 8.70 ^ab^ (7.31–29.19)	6.70 ± 4.20 ^b^ (2.42–16.39)	24.03 ± 23.40 ^a^ (1.17–66.28)
Glycine	63.78 ± 67.83 ^a^ (10.15–284.64)	28.35 ± 20.41 ^a^ (12.47–63.18)	42.61 ± 35.33 ^a^ (10.68–125.68)	68.55 ± 60.39 ^a^ (9.39–181.29)
DMS ^3^	3.44 ± 1.57 ^a^ (1.74–8.49)	4.15 ± 1.44 ^a^ (2.99–6.37)	0.99 ± 0.61 ^b^ (0.43–2.33)	3.80 ± 3.63 ^a^ (0.33–10.52)

^1^ TMA—trimethylamine; ^2^ DMA—dimethylacetamide; ^3^ DMS—dimethyl sulfide. ^4^ Lowercase letters indicate the comparisons among different countries (*p* < 0.05). ^5^ Values are presented as concentration ranges (minimum–maximum).

**Table 7:** Taste active values (TAVs) of various metabolites in fish sauces.

	Glucose	Betaine	Lactate	Gly	Val	Ala		Glucose	Betaine	Lactate	Gly	Val	Ala
Threshold ^1^	860 ^2^	250 ^2^	126 ^2^	130 ^3^	60 ^3^	40 ^3^	Threshold	860 ^2^	250 ^2^	126 ^2^	130 ^3^	60 ^3^	40 ^3^
Code	TAV						Code	TAV					
A1	1.08 *	0.04	0.38	**2.19**	**1.56**	**1.84**	B4	0.00	0.02	0.14	0.17	0.53	0.66
A2	0.34	0.02	0.20	**1.03**	0.80	0.94	B5	0.07	0.01	0.06	0.10	0.47	0.40
A3	0.68	0.02	0.19	0.31	0.66	0.79	C1	0.61	0.01	0.05	0.29	0.17	0.38
A4	0.28	0.02	0.16	0.62	0.65	0.71	C2	**1.33**	0.01	0.04	0.26	0.09	0.08
A5	0.57	0.02	0.21	0.61	0.67	0.85	C3	**1.20**	0.01	0.04	0.25	0.11	0.16
A6	0.60	0.02	0.21	0.26	0.79	0.83	C4	0.73	0.00	0.02	0.17	0.05	0.05
A7	0.78	0.01	0.13	0.24	0.42	0.53	C5	0.00	0.00	0.03	0.08	0.07	0.23
A8	0.06	0.01	0.08	0.10	0.52	0.46	C6	0.80	0.01	0.06	0.37	0.24	0.51
A9	0.07	0.01	0.11	0.13	0.72	0.63	C7	0.58	0.01	0.04	0.24	0.17	0.37
A10	0.61	0.02	0.18	0.23	0.61	0.74	D1	**1.84**	0.02	0.13	0.97	0.32	0.35
A11	0.85	0.02	0.21	0.36	0.77	0.90	D2	0.02	0.04	0.49	0.53	**1.92**	**2.42**
A12	0.54	0.02	0.16	0.49	0.67	0.75	D3	**3.42**	0.05	0.53	**1.39**	**1.62**	**2.19**
A13	0.30	0.01	0.11	0.35	0.44	0.46	D4	0.18	0.01	0.08	0.24	0.26	**1.08**
A14	0.48	0.01	0.09	0.15	0.28	0.34	D5	0.47	0.01	0.08	0.16	0.26	0.37
A15	0.48	0.00	0.03	0.08	0.10	0.11	D6	0.27	0.01	0.09	0.21	0.29	0.93
A16	0.21	0.01	0.12	0.71	0.48	0.58	D7	0.02	0.01	0.10	**1.31**	0.33	0.97
B1	0.06	0.04	0.23	0.49	**1.00**	**1.22**	D8	0.00	0.00	0.01	0.07	0.01	0.02
B2	0.05	0.02	0.08	0.12	0.49	0.59	D9	0.01	0.03	0.30	0.31	0.90	**1.42**
B3	0.01	0.02	0.10	0.22	0.65	0.76	D10	0.14	0.02	0.18	0.57	0.59	**1.40**

^1^ Unit as mg/100 mL. ^2^ Liu et al., 2018 [44]. ^3^ Wang et al., 2019 [45]. * TAVs > 1.00 are shown in bold.

## Data Availability

The original contributions presented in this study are included in the article; further inquiries can be directed to the corresponding author.

## Appendix A

**Table A1:** The ingredient English labeling in Thai and Korean fish sauce products.

Sample	Origin	Price TWD/100 mL	Raw Materials	Remark
A1	Thailand	50	Anchovies, salt, sucrose, syrup, licorrhizin	
A2	Thailand	45	Anchovies, salt, sugar, syrup	
A3	Thailand	25	Anchovies, sugar, salt	
A4	Thailand	25	Anchovies, salt, sugar	
A5	Thailand	80	Anchovies, salt, sugar	
A6	Thailand	11	Anchovies, salt, sugar	
A7	Thailand	20	Anchovies, salt, sugar	
A8	Thailand	25	Anchovies, salt, sugar	
A9	Thailand	22	Anchovies, salt, sugar	
A10	Thailand	24	Anchovies, salt, sugar	
A11	Thailand	11	Anchovies, salt, sugar	
A12	Thailand	9	Anchovies, iodized salt, sugar	
A13	Thailand	11	Anchovies, salt, sugar	
A14	Thailand	38	Anchovies, salt, sugar, monosodium glutamate (MSG)	
A15	Thailand	10	Gourami, *Anabas testudineus*, minnow, salt, sugar, sucralose, monosodium L-glutamate	
A16	Thailand	20	Anchovies, salt, sugar	
B1	Korea	18	Sand lance, salt	
B2	Korea	36	Sand lance, salt, shrimp, sugar, monosodium L-glutamate, sodium 5’-inosinate (IMP), sodium 5’-guanylate (GMP), maltodextrin, yeast extract, fructose, vitamin B1	
B3	Korea	24	Anchovies, salt, glucose, lactic acid, alcohol, IMP, GMP, glycine	
B4	Korea	24	Sand lance, shrimp, salt	
B5	Korea	24	Anchovies, salt, monosodium L-glutamate	
C1	Taiwan	7	Fish sauce, hydrolyzate of soybean protein, salt, sugar, disodium succinate, potassium sorbate	
C2	Taiwan	40	Mackerel fish sauce, salt, sucrose, potassium sorbate	
C3	Taiwan	20	Mackerel fish sauce, salt, sucrose	
C4	Taiwan	12	Mackerel fish sauce, salt, sucrose, potassium sorbate	
C5	Taiwan	7	Soybean hydrolyzate, fish sauce, salt, sucralose, acesulfame potassium, benzoic acid, silicon dioxide	
C6	Taiwan	8	Salt, anchovies, hydrolyzate of soybean protein, sugar	
C7	Taiwan	33	Milkfish, non-GMO soybeans, wheat, sugar, salt, rice vinegar, shiitake seasoning, IMP, sodium erythorbate	
D1	Vietnam	23	*Salanxchinensis*, salt, acesulfame potassium	
D2	Vietnam	7	Anchovies, salt, sugar, monosodium L-glutamate, GMP, IMP, acetic acid, citric acid, salmon flavor, potassium sorbate, sodium benzoate, xanthan gum, caramel color, carmine	
D3	Vietnam	10	Anchovies, salt, sugar, monosodium L-glutamate, DL-alanine, glycine, acetic acid, citric acid, salmon flavor, potassium sorbate, sodium benzoate, xanthan gum, aspartame, gardenia yellow, caramel color, carmine	TN > 10 g/L
D4	Vietnam	14	Anchovies, salt, sugar, monosodium L-glutamate, GMP, IMP, acetic acid, citric acid, flavor, potassium sorbate, sodium benzoate, xanthan gum, caramel color, carmine	
D5	Vietnam	14	Anchovies, salt, sugar, DL-alanine, monosodium L-glutamate, IMP, GMP, L-glutamic acid, glycine, acetic acid, citric acid, salmon flavor, sodium benzoate, xanthan gum, beta vulgaris color, caramel color, gardenia yellow, aspartame	
D6	Vietnam	16	Anchovies, iodized salt, L-glutamic acid, monosodium L-glutamate, IMP, GMP, DL-alanine, glycine, citric acid, sodium citrate, glucono-δ-lactone, fish sauce flavor, gardenia yellow	
D7	Vietnam	6	Salt, anchovies, monosodium L-glutamate, IMP, GMP, acetic acid, citric acid, sodium benzoate, potassium sorbate, acesulfame potassium, xanthan gum, caramel color	
D8	Vietnam	128	Anchovies, salt, E631(disodium inosinate), E950 (acesulfame potassium)	
D9	Vietnam	70	Anchovies, sugar, iodized salt, E330 (citric acid), D-xylitol, natural flavor, E950 (acesulfame potassium), caramel	TN = 35 g/L
D10	Vietnam	6	Anchovies, salt, monosodium L-glutamate, glycine, L-alanine, GMP, IMP, sodium citrate, DL-malic acid, soybean hydrolyzate, fish sauce flavor, salmon flavor, sodium benzoate, potassium sorbate, acesulfame potassium, aspartame, xanthan gum, caramel color, carmine, gardenia yellow	

## References

[1] Sanceda N.G., Kurata T., Arakawa N. (1996). Accelerated fermentation process for the manufacture of fish sauce using histidine. J. Food Sci..

[2] Gao P., Xia W., Li X., Liu S.Q. (2019). Use of wine and dairy yeasts as single starter cultures for flavor compound modification in fish sauce fermentation. Front. Microbiol..

[3] Grand View Research (2024). Fish Sauce Market Size, Share & Trends Analysis Report by Product, by Application, by Distribution Channel, by Region, and Segment Forecasts, 2024–2030.

[4] Tsai Y.H., Lin C.Y., Chien L.T., Lee T.M., Wei C.I., Hwang D.F. (2006). Histamine contents of fermented fish products in Taiwan and isolation of histamine-forming bacteria. Food Chem..

[5] Zarei M., Najafzadeh H., Eskandari M.H., Pashmforoush M., Enayati A., Gharibi D., Fazlara A. (2012). Chemical and microbial properties of mahyaveh, a traditional Iranian fish sauce. Food Control.

[6] Jiang W., Xu Y., Li C., Dong X., Wang D. (2014). Biogenic amines in commercially produced Yulu, a Chinese fermented fish sauce. Food Addit. Contam. Part B.

[7] Colombo F.M., Cattaneo P., Confalonieri E., Bernardi C. (2018). Histamine Food Poisonings: A Systematic Review and Meta-Analysis. Crit. Rev. Food Sci. Nutr..

[8] (2014). General Method of Test for Heavy Metals.

[9] Konosu S., Watanabe K., Shimizu T. (1974). Distribution of nitrogenous components in the muscle extracts of eight species of fish. Nippon. Suisan Gakkaishi.

[10] Cobb B., Alaniz I., Thompson C. (1973). Biochemical and microbial studies on shrimp: Volatile nitrogen and amino nitrogen analysis. J. Food Sci..

[11] Huang Y.R., Liu K.J., Hsieh H.S., Hsieh C.H., Hwang D.F., Tsai Y.H. (2010). Histamine level and histamine-forming bacteria in dried fish products sold in Penghu Island of Taiwan. Food Control.

[12] AOAC (2000). Official Method of Analysis.

[13] TISI (1983). Standard for Local Fish Sauce.

[14] Chen H.C., Huang Y.R., Hsu H.H., Lin C.S., Chen W.C., Lin C.M., Tsai Y.H. (2010). Determination of histamine and biogenic amines in fish cubes (*Tetrapturus angustirostris*) implicated in a food-borne poisoning. Food Control.

[15] Cho Y.J., Im S.Y., Lee K.W., Kim G.B., Choi Y.J. (1999). Quality investigation of commercial northern sand lance, *Ammodytes personatus* sauces. Korean J. Fish. Aquat. Sci..

[16] Um I.S., Park K.S. (2015). Biogenic amine contents of commercial salted and fermented sand lance *Ammodytes personatus* sauces. Korean J. Fish. Aquat. Sci..

[17] Park J.N., Fukumoto Y., Fujita E., Tanaka T., Washio T., Otsuka S., Shimizu T., Watanabe K., Abe H. (2001). Chemical composition of fish sauces produced in Southeast and East Asian countries. J. Food Compos. Anal..

[18] Nakano M., Sagane Y., Koizumi R., Nakazawa Y., Yamazaki M., Watanabe T., Takano K., Sato H. (2017). Data on the chemical properties of commercial fish sauce products. Data Brief.

[19] Lopetcharat K., Park J.W. (2002). Characteristics of fish sauce made from Pacific whiting and surimi by-products during fermentation stage. J. Food Sci..

[20] Yimdee T., Wang X.C. (2016). Comparison of odor and taste of commercial brand fish sauces from East and Southeast Asian countries. Int. J. Food Prop..

[21] (2022). Regulations on the Compliance of Nutritional Labeling on Packaged Foods.

[22] Zhu W., Luan H., Bu Y., Li J., Li X., Zhang Y. (2021). Changes in taste substances during fermentation of fish sauce and the correlation with protease activity. Food Res. Int..

[23] Bulushi I.A., Poole S., Deeth H.C., Dykes G.A. (2009). Biogenic amines in fish: Roles in intoxication, spoilage, and nitrosamine formation—A review. Crit. Rev. Food Sci. Nutr..

[24] International Agency for Research on Cancer (2012). IARC Monographs on the Evaluation of Carcinogenic Risks to Humans.

[25] Bekhit A.E.D.A., Holman B.W., Giteru S.G., Hopkins D.L. (2021). Total volatile basic nitrogen (TVB-N) and its role in meat spoilage: A review. Trends Food Sci. Technol..

[26] Park S., Sung J., Choi H.Y., Jeong J., Jeong H.G., Kim J.C., Jang M. (2023). Changes in the physicochemical properties and metabolites of *Myeolchi-jeot* (salted-fermented anchovy) based on fermentation time. LWT.

[27] Jiang J.J., Zeng Q.X., Zhu Z.W., Zhang L.Y. (2007). Chemical and sensory changes associated Yu-lu fermentation process—A traditional Chinese fish sauce. Food Chem..

[28] (2016). Korean Industrial Standards: Fermented Anchovy Sauce.

[29] Cho Y.J., Lee H.H., Kim B.K., Gye H.J., Jung W.Y., Shim K.B. (2014). Quality evaluation to determine the grading of commercial salt-fermented fish sauce in Korea. J. Fish. Mar. Sci. Educ..

[30] Joung B.C., Min J.G. (2018). Changes in postfermentation quality during the distribution process of anchovy (*Engraulis japonicus*) fish sauce. J. Food Prot..

[31] Moon J.S., Kim Y., Jang K.I., Cho K.J., Yang S.J., Yoon G.M., Kim S.Y., Han N.S. (2010). Analysis of biogenic amines in fermented fish products consumed in Korea. Food Sci. Biotechnol..

[32] Kim B.K., Kim Y.H., Lee H.H., Cho Y.J., Kim D.S., Oh S.M., Shim K.B. (2011). Comparison of the chemical compositions and biogenic amine contents of salt-fermented fish sauces produced in Korea to evaluate the quality characteristics. J. Fish. Mar. Educ..

[33] Kimura B., Konagaya Y., Fujii T. (2001). Histamine Formation by *Tetragenococcus muriaticus*, a Halophilic Lactic Acid Bacterium Isolated from Fish Sauce. Int. J. Food Microbiol..

[34] Kang H.W. (2013). Characteristics of kimchi added with anchovy sauce from heat and non-heat treatments. Culin. Sci. Hosp. Res..

[35] Tran Q.H., Nguyen T.T., Pham K.P. (2020). Development of the high sensitivity and selectivity method for the determination of histamine in fish and fish sauce from Vietnam by UPLC-MS/MS. Int. J. Anal. Chem..

[36] U.S. Food and Drug Administration (FDA) (2022). Fish and Fishery Products Hazards and Controls Guidance.

[37] European Commission (2013). Commission Regulation (EU) No 1019/2013 of 23 October 2013 amending Annex I to Regulation (EC) No 2073/2005 as regards histamine in fishery products. Off. J. Eur. Union.

[38] Wu T., Wang M., Wang P., Tian H., Zhan P. (2022). Advances in the formation and control methods of undesirable flavors in fish. Foods.

[39] Devos M.F.J.P. (1995). Standardized human olfactory thresholds. J. Odor Res. Eng..

[40] Van Gemert L.J. (2003). Compilations of Odour Threshold Values in Air, Water and Other Media.

[41] Udomsil N., Rodtong S., Choi Y.J., Hua Y., Yongsawatdigul J. (2011). Use of Tetragenococcus halophilus as a Starter Culture for Flavor Improvement in Fish Sauce Fermentation. J. Agric. Food Chem..

[42] Bai J., Baker S.M., Goodrich-Schneider R.M., Montazeri N., Sarnoski P.J. (2019). Aroma profile characterization of mahi-mahi and tuna for determining spoilage using purge and trap gas chromatography-mass spectrometry. J. Food Sci..

[43] Lapsongphon N., Yongsawatdigul J., Cadwallader K.R. (2015). Identification and characterization of the aroma-impact components of Thai fish sauce. J. Agric. Food Chem..

[44] Liu C., Meng F., Tang X., Shi Y., Wang A., Gu Z., Pan Z. (2018). Comparison of nonvolatile taste active compounds of wild and cultured mud crab *Scylla paramamosain*. Fish. Sci..

[45] Wang Y., Li C., Li L., Yang X., Chen S., Wu Y., Zhao Y., Wang J., Wei Y., Yang D. (2019). Application of UHPLC-Q/TOF-MS-based metabolomics in the evaluation of metabolites and taste quality of Chinese fish sauce (Yu-lu) during fermentation. Food Chem..

[46] McKelvie J.R., Yuk J., Xu Y., Simpson A.J., Simpson M.J. (2009). ^1^H NMR and GC/MS metabolomics of earthworm responses to sub-lethal DDT and endosulfan exposure. Metabolomics.

[47] Ciampa A., Laghi L., Picone G. (2022). Validation of a ^1^H-NMR spectroscopy quantitative method to quantify trimethylamine content and K-index value in different species of fish. J. Food Qual..

[48] Picone G. (2024). The ^1^H HR-NMR Methods for the Evaluation of the Stability, Quality, Authenticity, and Shelf Life of Foods. Encyclopedia.

